# Biodegradation of Uric Acid by *Bacillus paramycoides*-YC02

**DOI:** 10.3390/microorganisms11081989

**Published:** 2023-08-02

**Authors:** Xiaoyu Cao, Jingyuan Cai, Yu Zhang, Chao Liu, Meijie Song, Qianqian Xu, Yang Liu, Hai Yan

**Affiliations:** School of Chemistry and Biological Engineering, University of Science and Technology Beijing, Beijing 100083, China; d202210452@xs.ustb.edu.cn (X.C.); caijingyuan970317@163.com (J.C.);

**Keywords:** uric acid, *Bacillus paramycoides*-YC02, biodegradation, genomic analysis

## Abstract

High serum uric acid levels, known as hyperuricemia (HUA), are associated with an increased risk of developing gout, chronic kidney disease, cardiovascular disease, diabetes, and other metabolic syndromes. In this study, a promising bacterial strain capable of biodegrading uric acid (UA) was successfully isolated from *Baijiu* cellar mud using UA as the sole carbon and energy source. The bacterial strain was identified as *Bacillus paramycoides*-YC02 through 16S rDNA sequence analysis. Under optimal culture conditions at an initial pH of 7.0 and 38 °C, YC02 completely biodegraded an initial UA concentration of 500 mg/L within 48 h. Furthermore, cell-free extracts of YC02 were found to catalyze and remove UA. These results demonstrate the strong biodegradation ability of YC02 toward UA. To gain further insight into the mechanisms underlying UA biodegradation by YC02, the draft genome of YC02 was sequenced using Illumina HiSeq. Subsequent analysis revealed the presence of gene1779 and gene2008, which encode for riboflavin kinase, flavin mononucleotide adenylyl transferase, and flavin adenine dinucleotide (FAD)-dependent urate hydroxylase. This annotation was based on GO or the KEEG database. These enzymes play a crucial role in the metabolism pathway, converting vitamin B_2_ to FAD and subsequently converting UA to 5-hydroxyisourate (HIU) with the assistance of FAD. Notably, HIU undergoes a slow non-enzymatic breakdown into 2-oxo-4-hydroxy-4-carboxy-5-ureidoimidazoline (OHCU) and (S)-allantoin. The findings of this study provide valuable insights into the metabolism pathway of UA biodegradation by *B. paramycoides*-YC02 and offer a potential avenue for the development of bacterioactive drugs against HUA and gout.

## 1. Introduction

Uric acid (UA) is the end product of purine metabolism in humans with a molecular formula of C_5_H_4_N_4_O_3_ (7,9-dihydro-1H-purine-2,6,8(3H)-trione) and a molecular weight of 168.11 Da [1]. UA metabolism involves complex processes; under normal conditions, the production and excretion of UA in humans are basically in a dynamic balance. In humans, UA cannot undergo oxidative degradation to more soluble compound allantoin due to mutation in the gene coding for uricase [2]. The absence of uricase results in the excretion of UA, which is mainly divided into two ways, of which two-thirds is excreted through the kidneys and one-third is excreted through the intestines [3]. Hyperuricemia (HUA) is a metabolic disease owing to UA underexcretion, overproduction, or both, which is defined as a serum UA level above 7 mg/dl in males and 6 mg/dl in females [4]. The global prevalence of HUA has increased significantly in recent years, with an overall trend of increase and rejuvenation [5]. The latest data showed that the prevalence of HUA in mainland China was 17.4%, and the prevalence in men was twice as much as in women [6]. Another survey revealed that the prevalence of HUA in the U.S. was 20.2% in males and 20.0% in females [7]. Early HUA has no clinical symptoms and is characterized only by elevated serum UA levels, but with the development of the disease, it may lead to gout [8], chronic kidney disease [9], cardiovascular disease [10], hypertension [11], type 2 diabetes mellitus [12] and other metabolic syndromes [13]. Therefore, the development of effective methods to manage HUA become a hotspot of current biomedical research.

Currently, the management of HUA involves pharmacological treatment and dietary intervention. Chemical drugs used for HUA treatment can be classified into three main categories: xanthine oxidase (XOD) inhibitors (such as allopurinol, febuxostat, and topiroxostat), uricosuric agents (such as lesinurad, probenecid, and benzbromarone), and enzyme therapies (such as rasburicase and pegloticase) [14]. Their mechanisms of action include decreasing UA production, increasing UA excretion and metabolizing serum UA to allantoin. Exogenous medications are effective, but long-term use can cause varying degrees of damage to the body and can lead to reduced efficacy and allergic reactions [15]. For example, a report shows that benzbromarone has adverse effects on liver and kidneys function, which had been withdrawn from the most part of countries [16]. Another report shows allopurinol may cause mild rashes and severe skin reactions [17]. Although several novel drugs, including Ulodesine (an inhibitor of purine nucleoside phosphorylase), RLBN1001, and KUX-1511 (inhibitors of XOD and urate transporter 1) [18], are currently under development, the management of HUA still faces suboptimal outcomes.

In addition, traditional Chinese medicine has been used to manage HUA and gout from a long time ago, such as Simiao Powder (*Phellodendri amurensis cortex*, *Semen coicis*, *Radix achyranthes root*, *Atractylodis rhizoma*) [19], Compound Tufuling Granules [20], *Astragalus membranaceus* [21], *Sanghuangporus vaninii* and *Inonotus hispidus* [22]. Chinese herbal medicines possess intricate compositions and exert their effects on multiple targets to reduce serum UA levels. They achieve this by targeting the UA transporter, inhibiting UA synthesis, alleviating inflammation, guarding against renal fibrosis, and modulating oxidative stress [23]. Moreover, gut microbiota can be modulated, and the abundances of beneficial bacterium can be increased by Chinese herbal medicines [24]. Compared to western medicine in the management of HUA, the unclear molecular mechanism of action greatly limits their clinical applicability [25].

In recent decades, an increasing number of studies have demonstrated that diet plays a crucial role in the development of HUA and gout. However, dietary interventions necessitate restricting the consumption of purine-rich foods, alcohol, and fructose. The recommended daily intake of purines in the diet is less than 400 mg in Japan. Excessive intake of purine-rich foods can lead to elevated serum UA levels [26], which is mainly due to the fact that exogenous purines are basically converted into UA in the human body. An excessive intake of alcohol and fructose can cause decreased UA excretion, increased UA production, or both. The primary reasons are their potential to affect the kidneys’ normal excretion of UA and their dependence on substantial quantities of adenosine triphosphate (ATP) and phosphate for liver metabolism [27]. Undoubtedly, dietary intervention holds great significance not only in terms of economic considerations but also due to the potential adverse effects associated with pharmacological treatment. However, dietary interventions necessitate significant patient cooperation and long-term adherence [28], which often leads to low patient compliance.

According to the available research, the microbial biodegradation of UA provides new research ideas for the treatment of HUA and gout. Some studies showed that lactic acid bacteria (LAB) could biodegrade UA and had the potential to intervene in the treatment of HUA. These LAB were isolated from various fermented foods, such as *Lactobacillus plantarum* Q7 (from yak yogurt) [29] and *Limosilactobacillus fermentum* JL-3 (from *Jiangshui*) [30]. But more studies about the amelioration of HUA by LAB focus on degrading purine compounds [31] and suppressing XOD activity [32]; many LAB do not have the ability to biodegrade UA directly. Others indicated that some *Bacillus* had the ability to produce uricase, including *B. subtillis* [33], *B. licheniformis* [34], *B. thermocatenulatus* [35] and *B. cereus* [36], but the ability of all bacteria reported in the biodegradation of UA was low.

In this study, a bacterial strain that has a better ability in UA’s biodegradation is first isolated from *Baijiu* cellar mud and identified as *Bacillus paramycoides*-YC02. The culture conditions were optimized, and both YC02 and its cell-free extract (CE) demonstrated the effective removal of UA, indicating the production of UA-biodegrading enzymes by YC02. Subsequently, the draft genome of YC02 was sequenced to find genes encoding UA biodegradation enzymes, leading to the elucidation of the biodegradation mechanism. These findings hold significant importance in the development of bacterioactive drugs targeting HUA and gout.

## 2. Materials and Methods

### 2.1. Samples and Mediums

UA with a purity of 99% was purchased from Aladdin Chemical Co. (Rogers, MN, USA), and all other chemicals used in this study were analytical grade. A bacterial strain used in this study was isolated from *Baijiu* cellar mud of Shandong Bandaojing Co., Ltd. (Gaoqing County, China) in China using UA as the sole carbon and energy source.

The modified mineral salt medium (MSM) for the isolation of bacteria contained (g/L): NH_4_Cl, 0.5; vitamin B complex tablets, 0.01; Na_2_HPO_4_, 0.5; KH_2_PO_4_, 0.05; MgSO_4_, 0.1; CaCl_2_, 0.01; C_6_H_11_FeNO_7_, 0.01; trace element solution 0.5 mL and H_2_O 1 L. UA at different concentrations as the sole carbon and energy source were added in the modified MSM. The Luria–Bertani (LB) medium for culture contained (g/L): NaCl, 10; Peptones, 10; Glucose, 5; Yeast powder, 5 and H_2_O 1 L. The initial pH of the modified MSM and LB medium was adjusted to 7.0 using 0.1 M NaOH solution. Both were sterilized in an autoclave (LDZH-100L, Shanghai, China) at 121 °C for 20 min.

### 2.2. Isolation of UA Biodegrading Bacteria

Ten grams of *Baijiu* cellar mud samples were added to 100 mL of sterilized water, stirred thoroughly and left for 30 min; then, 5 mL of supernatant was taken into a 250 mL flask that contained 45 mL of modified UA-MSM and incubated for 7 days at 38 °C with the shake rate of 200 rpm in an incubator shaker. Every 7 days, 5 mL of the cultures was subcultured to fresh modified UA-MSM with the same culture conditions each time. The concentration of UA was increased from 0.5 to 4 g/L (0.5, 1, 2, 3, 4 g/L) [37]. After 5 weeks, the last cultures were continuously diluted and then inoculated with the spread method onto the LB agar plates and incubated for 48 h at 38 °C. Single colonies grown on the LB agar plates were picked and inoculated into modified MSM containing UA to test biodegradation abilities, which were repeated several times until a pure bacterial strain was isolated.

### 2.3. Identification and Draft Genome Sequencing of YC02

The morphology of YC02 was observed by a microscope (CX41, Olympus, Tokyo, Japan). The YC02 was inoculated in LB medium and incubated for 48 h at 38 °C with the shake rate of 200 rpm. Then, 5 mL cultures of YC02 were used to extract genomic DNA with a bacterial genomic DNA kit. By using the extracted bacterial genome as the template, a pair of universal primers 27F (5′-AGAGTTTGATCCTGGCTCAG-3′) and 1492R (5′-GGTTACCTTGTTACGACTT-3′) were used to perform PCR [38]. The purified PCR products were sent to the Biotechnology Co. (Shanghai, China) for sequencing. The 16S rDNA sequences of selected strains were analyzed by BLAST comparison through GenBank and the Ribosomal Database Project. The phylogenetic tree was constructed based on the 16S rDNA gene sequences using the neighbor-joining method by MEGA 6.0.

The culture solution of YC02 was prepared, and the bacterial precipitate was collected by the centrifugation of 10,000 rpm for 20 min at 4 °C, which was sent to Meiji Bio Co. (Shanghai, China) for sequencing the draft genome [39].

### 2.4. Optimization of UA Biodegradation Conditions

The biodegradation experiments by YC02 were carried out in 20 mL sterilized modified MSM containing 500 mg/L UA and grown at 38 °C with the shake rate of 200 rpm for 72 h. The optical density (OD_600nm_) was measured to represent the bacterial growth. In this experiment, three factors of initial pH, temperature and initial UA concentration were tested as independent variables to investigate their effects on the biodegradation ratios of UA by YC02. The initial pH of modified MSM was set at 5.0, 6.0, 7.0, 8.0 and 9.0 (38 °C, initial concentration of UA at 500 mg/L); temperature at 20, 25, 30, 38 and 40 °C (initial pH 7.0, initial concentration of UA at 500 mg/L); and initial concentrations of UA at 100, 200, 500, 1000 and 1500 mg/L (initial pH 7.0, 38 °C). Every 12 h, 2 mL of cultures was taken for measuring UA concentration and OD_600nm_.

The corresponding results were calculated by the following formula:

Biodegradation ratio (%) = (C0 − Ct)/C0 × 100%

C0—Initial concentration of UA, mg/L;

Ct—Residual concentration of UA in the sample at time t, mg/L.

### 2.5. Biodegradation of UA by CE of YC02

The YC02 was inoculated in 20 mL sterilized LB medium and incubated for 48 h at 38 °C with the shake rate of 200 rpm. Then, the cultures of YC02 were centrifuged at 12,000 rpm for 20 min [40]. The sediments of YC02 cells were washed several times, re-suspended with sterilized phosphate-buffered solution PBS (pH 7.0), and then ultrasonicated for 25 min at 450 W [41]. The supernatant as CE was obtained by centrifugation at 10,000 rpm for 20 min at 4 °C. Then, 5 mL of CE was added to sterilized PBS (pH 7.0), with a UA concentration of 570 mg/L. The reaction was carried out at 38 °C with the shake rate of 200 rpm for 12 h. Afterwards, 0.5 mL of cultures was taken at 0, 1, 2, 5, 7, 9, 12 h for measuring UA concentration, respectively. The concentration of protein was determined with the BCA method [42].

### 2.6. Analysis of UA by HPLC

UA was measured by HPLC (Shimadzu LC-20AT, Tokyo, Japan) [43]. The sample tested was diluted a certain number of times using 0.5 M NaOH solution and centrifuged at 12,000 rpm for 20 min. The supernatant passed through an aqueous phase microporous membrane (0.22 μm) and was used to detect concentration levels. Then, 20 µL mixed solution was extracted and analyzed by HPLC (chromatographic column: Kromasil C18 (4.6 × 250 mm, 5 μm-Micron); UV detection wavelength: 283 nm; mobile phase: methanol: 0.5% acetic acid aqueous solution (10:90); flow rate: 1 mL/min; column temperature: 35 °C). The standard curve between the content and peak area of UA was established, which was used to calculate the concentration of UA with peak area. Each sample was measured thrice; then, the results were averaged and marked with the standard deviation to represent UA concentration.

## 3. Results and Discussion

### 3.1. Isolation and Identification of UA Biodegrading Strain

**Figure 1:**
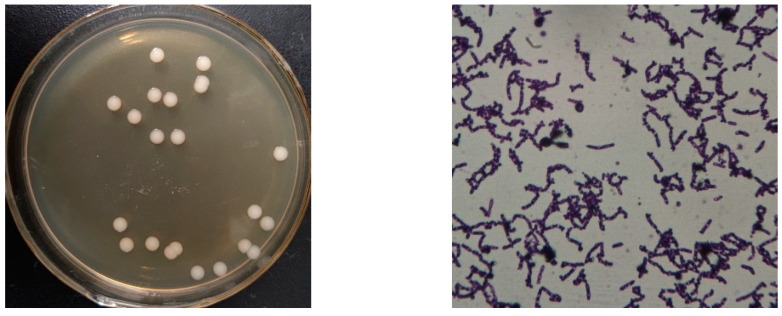
Colonies were grown on LB agar plate (**left**) and morphology (1000×) under the microscope (**right**) of YC02.

The monoclonal colonies of YC02 were grown on the LB agar plate (Figure 1 left), which indicates the slightly shiny white color of YC02 colonies. YC02 is Gram-positive, and the rod-shaped cells and the central spores were observed under a light microscope with 1000× magnification (Figure 1 right), which is preserved in China General Microbiological Culture Collection Center (CGMCC NO: 22812).

**Figure 2:**
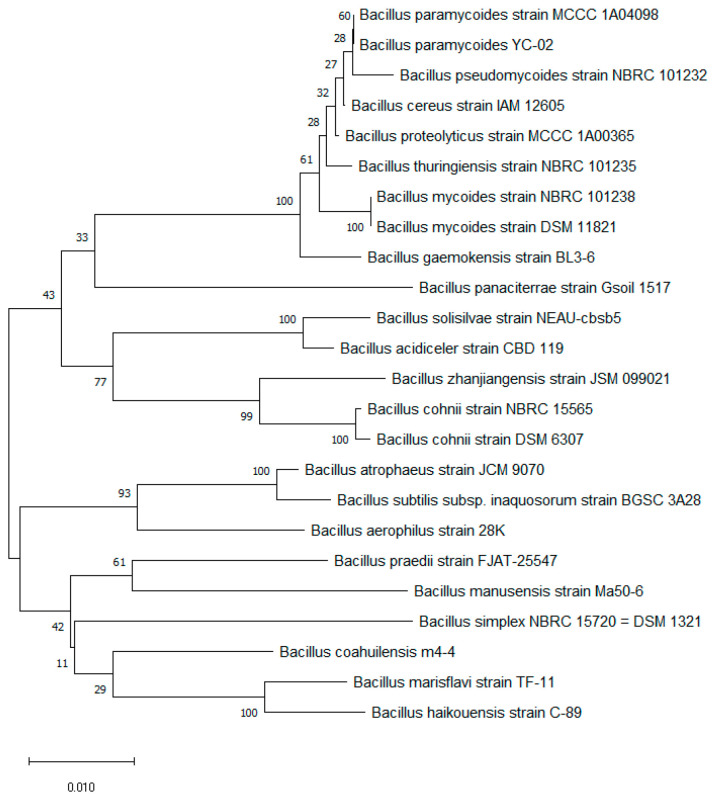
Phylogenetic tree of *B. paramycoides*-YC02.

According to Figure 2, the association between YC02 and other closely related members reveals YC02’s closest resemblance to *Bacillus paramycoides*, which was identified as a new species within the *B. cereus* group in 2017 by Yang Liu et al. [44]. The strain was identified as *B. paramycoides*-YC02 based on phylogenetic analysis of the 16S rDNA sequence. *B. paramycoides* was reported to biodegrade many organics. For example, a report showed that *B. paramycoides* could first degrade acephate to methamidophos, and then methamidophos was degraded to some small molecules by this bacterium [45]. Another report showed that *B. paramycoides* could utilize polyethylene as the sole carbon source [46]. In addition, it had been reported that *B. paramycoides* could produce a variety of enzymes [47,48,49], such as alpha-amylase, alkali thermos tolerant xylanase and ligninolytic enzyme. It also had the prospect of applications in soil remediation [50] and wastewater treatment [51]. To the best of our knowledge, no previous reports regarding UA biodegradation by *B. paramycoides* have been documented.

### 3.2. Effects of Culture Conditions on Biodegradation of UA by YC02

**Figure 3:**
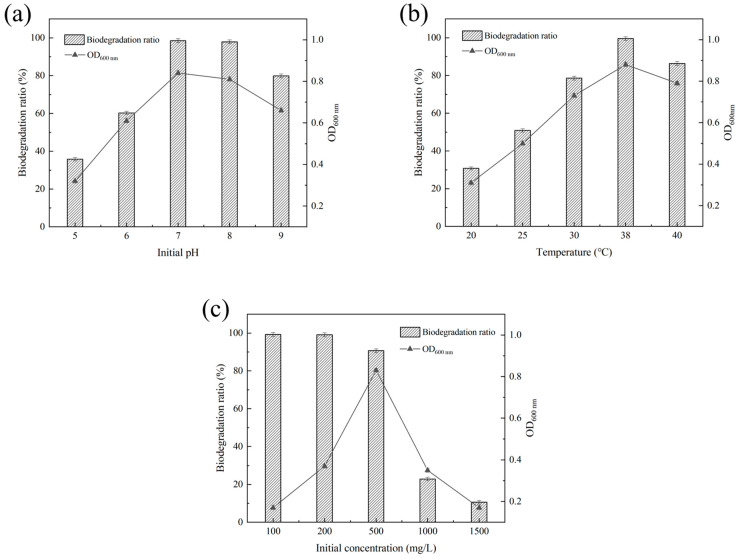
Effects of initial pH (**a**), temperature (**b**), and initial concentrations of UA (**c**) on growth and UA biodegradation ratio by YC02 cultured for 72 h.

The effects of different initial pH, temperature and initial concentration on the growth and the biodegradation ratios of UA by YC02 were investigated. UA biodegradation ratios exceeded 97% at initial pH values of 7.0 and 8.0, with no significant difference observed in YC02 growth (Figure 3a). These results show that the neutral and slightly alkaline conditions were favorable for the UA biodegradation by YC02, and the acidic environment would inhibit the growth. Consequently, the optimal biodegradation initial pH was determined to 7.0. Conversely, UA biodegradation ratios were significantly different among tested temperatures in range of 20–40 °C (Figure 3b), and it was 99.6% at 38 °C but declined to 30.8%, 50.9%, 78.6% and 86.4% when the culture temperature was 20, 25, 30 and 40 °C, respectively. These findings suggest that the optimal temperature for UA biodegradation is 38 °C. Furthermore, when the initial concentration of UA was below 500 mg/L, YC02 demonstrated biodegradation ratios exceeding 90.6%. However, as the initial UA concentration increased to 1000 mg/L and 1500 mg/L, the biodegradation ratios decreased to 22.8% and 10.6%, respectively (Figure 3c). These results indicate that both lower and higher initial UA concentrations limit and inhibit the growth of YC02. Consequently, the subsequent biodegradation experiments by YC02 employed the optimal culture conditions of initial pH 7.0 and 38 °C.

### 3.3. Biodegradation of UA by B. Paramycoides-YC02 and Its CE

**Figure 4:**
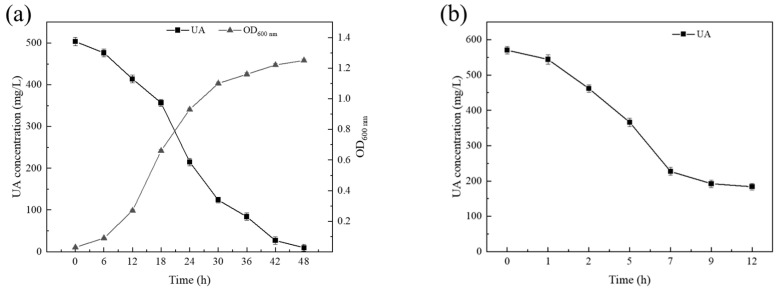
Growth and biodegradation of UA by YC02 (**a**) and its CE (**b**).

*B. paramycoides*-YC02 could completely remove 500 mg/L UA within 48 h; meanwhile, the OD_600nm_ could reach above 1.2 and the logarithmic growth period of YC02 began at 12 h (Figure 4a). These results indicate that YC02 has a better ability in the biodegradation of UA than any other bacteria reported. The in vitro biodegradation of UA with *L. plantarum* Q7 had been studied by Jiayuan Cao et al. [29]. The biodegradation ratio could reach 81.30%. Moreover, the biodegradation ratios of nucleotides, nucleosides and purine by *L. plantarum* Q7 were 99.97%, 99.15% and 87.35%, respectively. The in vivo biodegradation of UA with *Limosilactobacillus fermentum* JL-3 had also been studied by Ying Wu et al. [30]. After 15 days of intervention, JL-3 group could decrease UA levels in hyperuricemic mice, as the results showed that the UA levels significantly decreased to more than 30% in feces and urine. These reports have implications for our follow-up biodegradation experiments, and UA’s biodegradation experiment in vivo needs to be performed.

The CE of *B. paramycoides*-YC02 containing a protein concentration of 5.38 g/L could remove 570 mg/L of UA to 183 mg/L within 12 h (Figure 4b). In general, upon increasing the protein concentration of CE, the biodegradation ability is greatly improved [52]. The intracellular crude enzyme of YC02 exhibited high activity for up to 7 h, after which the enzyme activity gradually diminished. These results confirm YC02’s capability to produce enzymes that facilitate the biodegradation of UA. However, despite this study’s efforts, no discernible products were observed during the biodegradation of UA by *B. paramycoides*-YC02.

### 3.4. Genomic Analysis and Metabolism Pathway for UA Biodegradation

To delineate the mechanism of UA removed by YC02 treatment, the draft genome was sequenced using the Illumina Hiseq platform with paired-ends sequencing. It revealed a total length of 5,487,337 bp, with an average GC content of 35.14%. The reads were assembled into 66 scaffolds with an N50 of 641,208 bp, and 5609 protein-coding genes, 105 tRNA genes, 9 rRNA genes and 140 sRNA genes were predicted.

**Figure 5:**
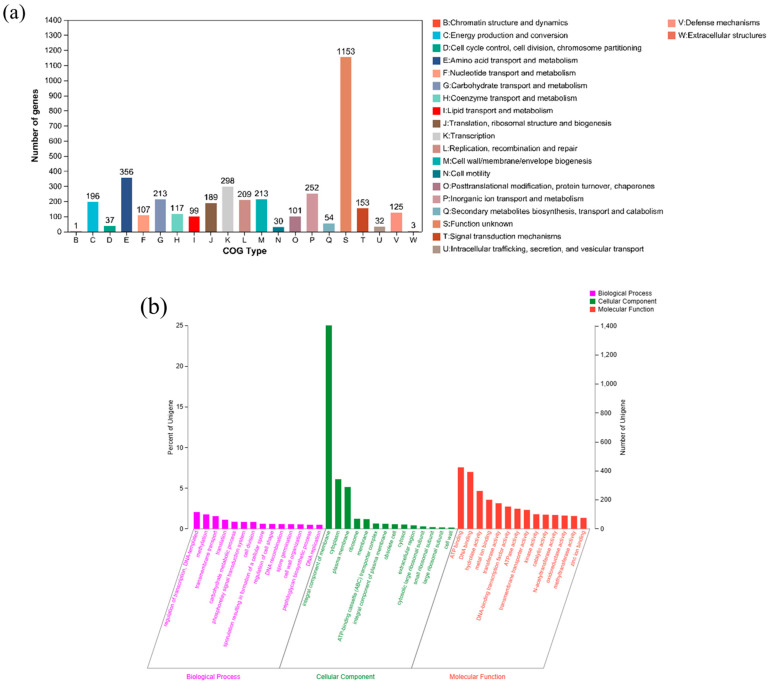

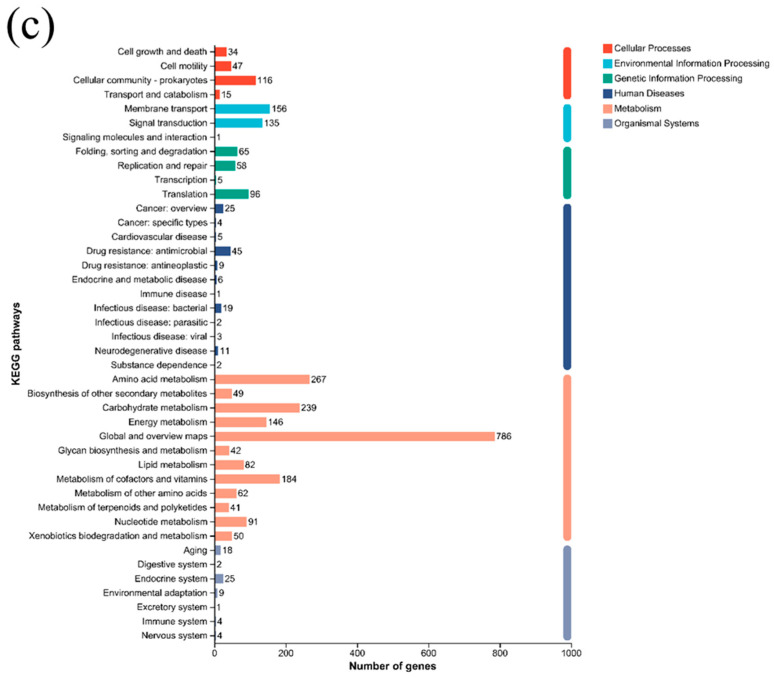
COG (**a**), GO (**b**), KEEG (**c**) annotation classification of *B. paramycoides*-YC02.

The result of genome annotation revealed that 69.19% of genes (3881) were categorized into 21 different categories of COG (Figure 5a). Notably, 213 genes were associated with carbohydrates transport and metabolism (G), 356 genes were associated with amino acid transport and metabolism (E), 298 genes were associated with transcription (K), and 252 genes were associated with inorganic ion transport and metabolism (P). Furthermore, 4118 genes were annotated in the GO database, with 76.1% of genes (3135) attributed to molecular function (Figure 5b). The genes’ proportion of ATP binding, DNA binding, hydrolase activity, metal ion binding and transferase activity were 7.58%, 7.01%, 4.67%, 3.58% and 3.16%, respectively. Additionally, 2538 genes were annotated in the KEGG database, and among them, 786 genes were associated with the general metabolic pathway, 267 genes were associated with amino acid metabolism, 239 genes were associated with carbohydrate metabolism, and 184 genes were associated with cofactors and vitamins metabolism (Figure 5c).

**Figure 6:**
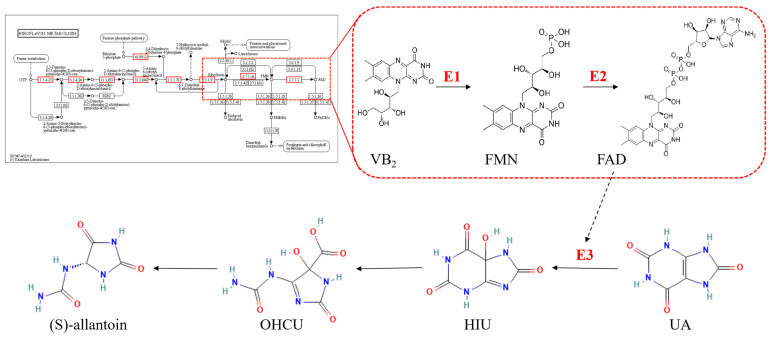
The metabolism pathway for biodegrading UA by *B. paramycoides*-YC02, E1: riboflavin kinase; E2: FMN adenylyl transferase; E3: FAD-dependent urate hydroxylase.

In the present study, *B. paramycoides*-YC02 was found to be able to biodegrade UA. According to the results mentioned above, the genes and enzymes involved in the direct conversion of UA to allantoin were not found. But the flavin adenine dinucleotide (FAD)-dependent urate hydroxylaseen encoded by gene2008 was found in the GO database. Meanwhile, riboflavin kinase and flavin mononucleotide (FMN) adenylyl transferase encoded by gene1779 were found in the KEEG database. Based on these results, UA’s biodegradation pathway was identified (Figure 6). Firstly, vitamin B_2_ was converted to FMN by riboflavin kinase, and then, it was converted to FAD by FMN adenylyl transferase [53]. Afterwards, FAD-dependent urate hydroxylase converted urate to 5-hydroxyisourate (HIU) with the assistance of FAD [54]. However, HIU could be spontaneously broken down to 2-oxo-4-hydroxy-4-carboxy-5-ureidoimidazoline (OHCU) and (S)-allantoin in vitro at a slow non-enzymatic rate [55]. This is consistent with the general pathway of UA, which is catabolized by bacteria.

During the genomic analysis, apart from gene 1779 and gene 2008, we also identified additional genes capable of encoding enzymes involved in the purine metabolism pathway in humans (Table 1). These enzymes have the biodegrading ability on the precursors of UA synthesis for reducing the amount of UA production. In the past report, the whole genome sequencing of *L. brevis* DM9218 was performed by Haina Wang et al., the gene named ORF00084 was discovered, and they verified the inosine hydrolyzing ability of the gene product. The gene product was an inosine hydrolase, which can decrease UA production by degrading the inosine that is the most important precursor of UA synthesis [56]. Next, we will proceed to validate the biodegradation capability of inosine and guanosine by YC02 and its CE. Upon obtaining these results, we will initiate the cloning of genes from the YC02 genome and the construction of engineered bacteria for application in a rat model of HUA [57].

At present, fermented Chinese herbal medicines with microorganisms for the management of HUA is also a focus of our current research. Ruoyu Wang et al. found that *B. subtilis* fermented *Astragalus membranaceus* (BFA) could decrease the serum UA levels in hyperuricemic mice [58]. Recently, it has been reported that inflammation can affect the expression of UA transporters in UA reabsorption, which results in elevated serum UA levels [59]. BFA could attenuate renal inflammation and regulate the expression of urate transporters. Not only that, BFA could enhance the gut barrier and restore gut microbiota. This report indicates that Chinese herbal medicines fermented with microorganisms have the potential to become a novel functional food for ameliorating HUA. In this study, we also want to know whether YC02 has the potential to ferment Chinese herbal medicine. However, in CAZy annotation, 119 genes encoding carbohydrate-active enzymes were founded, including Glycosyl Transferases (43 genes), Carbohydrate Esterases (38 genes), Glycoside Hydrolases (43 genes), Auxiliary Activities (13 genes) and Polysaccharide Lyases (1 gene). According to CAZy annotation, the fermentation of Chinese traditional herbal medicines by *B. paramycoides*-YC02 has potential promising applications in the prevention and treatment of HUA. In the following research, we also want to explore the potential therapeutic ability of *B. paramycoides*-fermented different Chinese herbal medicines on HUA, especially homologous medicines and foods (such as *Poria cocos* (Schw.) wolf, *Polygonatum mill* and *Astragalus membranaceus*).

## 4. Conclusions

In this study, we firstly isolated *B. paramycoides*-YC02, an efficient bacterium for biodegrading UA, from *Baijiu* cellar mud. Under the optimal culture conditions (initial pH 7.0, 38 °C), YC02 completely biodegraded an initial UA concentration of 500 mg/L within 48 h. Moreover, the CE of YC02, containing 5.38 g/L protein, successfully removed 387 mg/L of UA within 12 h. These findings clearly demonstrate YC02’s remarkable capacity for UA biodegradation. Importantly, the draft genome analysis revealed the presence of gene1779 and gene 2008, encoding riboflavin kinase, FMN adenylyl transferase and FAD-dependent urate hydroxylase, which are involved in UA biodegradation. Notably, FAD-dependent urate hydroxylase plays a crucial role in the biodegradation process by converting urate to HIU with the assistance of FAD. Subsequently, HIU spontaneously breaks down into OHCU and (S)-allantoin. These findings shed light on the metabolic pathway employed by YC02 in UA biodegradation. Additionally, our investigation unveiled numerous genes that encode enzymes responsible for the biodegradation of UA precursors and carbohydrate-active enzymes. These findings open up new avenues for future research on decreasing serum UA levels, including the biodegradation of inosine, guanosine and XOD inhibition. Not only that, we can also combine YC02 with Chinese herbal medicines to develop functional foods. We anticipate that these results will provide valuable insights into the amelioration of HUA and gout.

## Figures and Tables

**Table 1:** Genes and corresponding enzymes related to purines’ biodegradation in YC-02.

Gene ID	Database	Enzyme
gene 1530, gene 2198	KEGG	purine nucleosidase (EC:3.2.2.1)
gene 2643, gene 4968	COG	nucleoside hydrolase
gene 3823, gene 4212	KEGG	5’-nucleotidase (EC:3.1.3.5)
gene 4226	KEGG	purine-nucleoside phosphorylase (EC:2.4.2.1)
gene 2891, gene 5313	KEGG	hypoxanthine phosphoribosyltransferase (EC:2.4.2.8)
gene 0649	KEGG	xanthine phosphoribosyltransferase (EC:2.4.2.22)
gene 3326	KEGG	adenine phosphoribosyltransferase (EC:2.4.2.7)
gene 2775	KEGG	adenine deaminase (EC:3.5.4.2)

## Data Availability

Not applicable.
